# Sex differences in type 2 diabetes

**DOI:** 10.1007/s00125-023-05891-x

**Published:** 2023-03-10

**Authors:** Alexandra Kautzky-Willer, Michael Leutner, Jürgen Harreiter

**Affiliations:** 1 Department of Medicine III, Division of Endocrinology and Metabolism, Medical University of Vienna, Vienna, Austria; 2 Gender Institute, Lapura Women’s Health Resort, Gars am Kamp, Austria

**Keywords:** Cardiovascular mortality, Gender, Macrovascular complications, Microvascular complications, Review, Risk factors, Sex, Therapy, Type 2 diabetes

## Abstract

The prevalence of type 2 diabetes mellitus is increasing in both sexes, but men are usually diagnosed at a younger age and lower body fat mass than women. Worldwide, an estimated 17.7 million more men than women have diabetes mellitus. Women appear to bear a greater risk factor burden at the time of their type 2 diabetes diagnosis, especially obesity. Moreover, psychosocial stress might play a more prominent role in diabetes risk in women. Across their lifespan, women experience greater hormone fluctuations and body changes due to reproductive factors than men. Pregnancies can unmask pre-existing metabolic abnormalities, resulting in the diagnosis of gestational diabetes, which appears to be the most prominent risk factor for progression to type 2 diabetes in women. Additionally, menopause increases women’s cardiometabolic risk profile. Due to the progressive rise in obesity, there is a global increase in women with pregestational type 2 diabetes, often with inadequate preconceptual care. There are differences between men and women regarding type 2 diabetes and other cardiovascular risk factors with respect to comorbidities, the manifestation of complications and the initiation of and adherence to therapy. Women with type 2 diabetes show greater relative risk of CVD and mortality than men. Moreover, young women with type 2 diabetes are currently less likely than men to receive the treatment and CVD risk reduction recommended by guidelines. Current medical recommendations do not provide information on sex-specific or gender-sensitive prevention strategies and management. Thus, more research on sex differences, including the underlying mechanisms, is necessary to increase the evidence in the future. Nonetheless, intensified efforts to screen for glucose metabolism disorders and other cardiovascular risk factors, as well as the early establishment of prophylactic measures and aggressive risk management strategies, are still required for both men and women at increased risk of type 2 diabetes. In this narrative review we aim to summarise sex-specific clinical features and differences between women and men with type 2 diabetes into risk factors, screening, diagnosis, complications and treatment.

**Graphical abstract:**

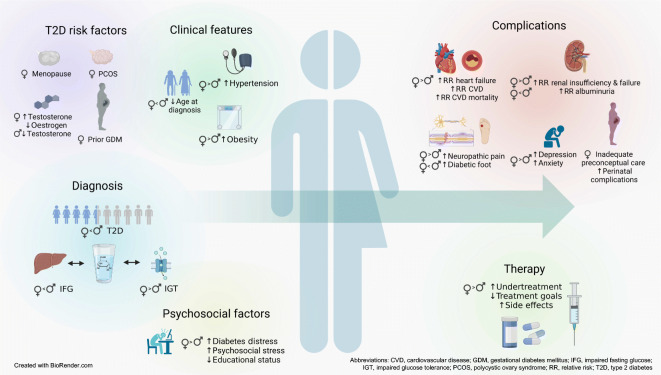

**Supplementary Information:**

The online version contains a slideset of the figures for download available at 10.1007/s00125-023-05891-x.

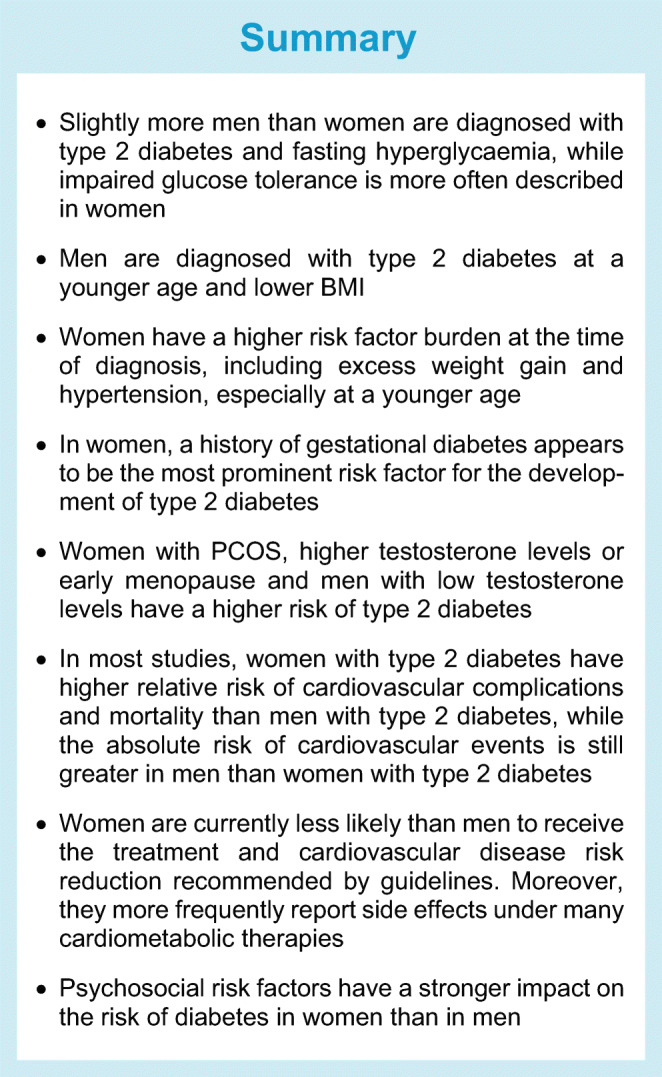



## Introduction

In young and middle-aged populations, men show a higher prevalence of type 2 diabetes mellitus than women [1]. However, postprandial hyperglycaemia increases to a larger extent in women as they age, contributing to a higher prevalence of undiagnosed diabetes in women after the age of 60, and of total diabetes after 70 [2]. Although the burden remains substantial, an improvement in life expectancy for patients with type 2 diabetes was recently reported for both sexes [3]. Lifetime risk of type 2 diabetes was generally higher in men, but years of life lost varied strongly between regions and sexes.

There is a lifelong continuous interaction between biology and environment, beginning in utero [4]. Biological ‘sex differences’ in the clinical outcomes of type 2 diabetes are caused by genetic and hormonal influences on pathophysiology, clinical manifestation, diagnosis and response to therapy [4, 5]. Across their lifetime, changes in sex hormones mean that women experience greater variations in the risk of cardiometabolic disease, including type 2 diabetes. Furthermore, ‘gender differences’ arising from psychosociocultural processes, such as different behaviours, lifestyles and attitudes towards prevention and treatment, also impact the susceptibility and progression of type 2 diabetes [4–6].

This narrative review is clinically oriented and aims to increase clinicians’ and researchers’ awareness of the differences between men and women in the risk, diagnosis and therapy of type 2 diabetes and its related complications, with the hope of improving management of all patients with type 2 diabetes.

The PubMed database was searched for full-text articles published between 1 January 2011 and 31 August 2022. The search terms used were ‘sex’ or ‘gender’ in combination with ‘diabetes’ in the title. The selection was limited to human studies and type 2 diabetes. All results were screened for relevant articles. Authors contributed additional articles based on their personal knowledge.

## Risk factors

### Insulin resistance

Studies have provided evidence that premenopausal women have higher skeletal muscle and hepatic insulin sensitivity and higher stimulated insulin secretion, and thus lower fasting glucose and HbA_1c_ values, than men [4, 7]. However, at menopause, BP, LDL-cholesterol and HbA_1c_ increase in parallel with unfavourable changes in body fat distribution [4], contributing to impaired glucose tolerance (IGT). With the progression from normal glucose tolerance to IGT, the biological advantages of women are mitigated [4]. Older women who were normoglycaemic were shown to have a ~20% higher glucagon-like peptide-1 (GLP-1) response to an OGTT compared with men of a similar age [8]. However, in the presence of IGT, impaired fasting glucose (IFG) or type 2 diabetes, women showed lower GLP-1 release than men, again suggesting that as glucose tolerance worsens, sex differences benefiting women disappear [8]. Notably, in the presence of overt type 2 diabetes, young women display cardiovascular- and total mortality risks comparable to men [4, 9]. Indeed, studies have shown that before the onset of type 2 diabetes, women have a greater exposure to, and burden of, major metabolic risk factors, such as greater changes in BMI, BP, fasting glucose and lipids [10, 11].

Notably, non-alcoholic fatty liver disease (NAFLD) diagnosis improves the risk prediction of type 2 diabetes, especially in premenopausal women [12]. As such, severe NAFLD is strongly and independently associated with incident type 2 diabetes in younger women, showing that NAFLD accentuates the loss of biological protection from type 2 diabetes in women. Indeed, women with dysglycaemia displayed a higher probability of having NAFLD than men, possibly related to a greater worsening of metabolic risk factors along with deterioration of glucose metabolism in women [13].

### Obesity and body fat distribution

**Fig. 1:**
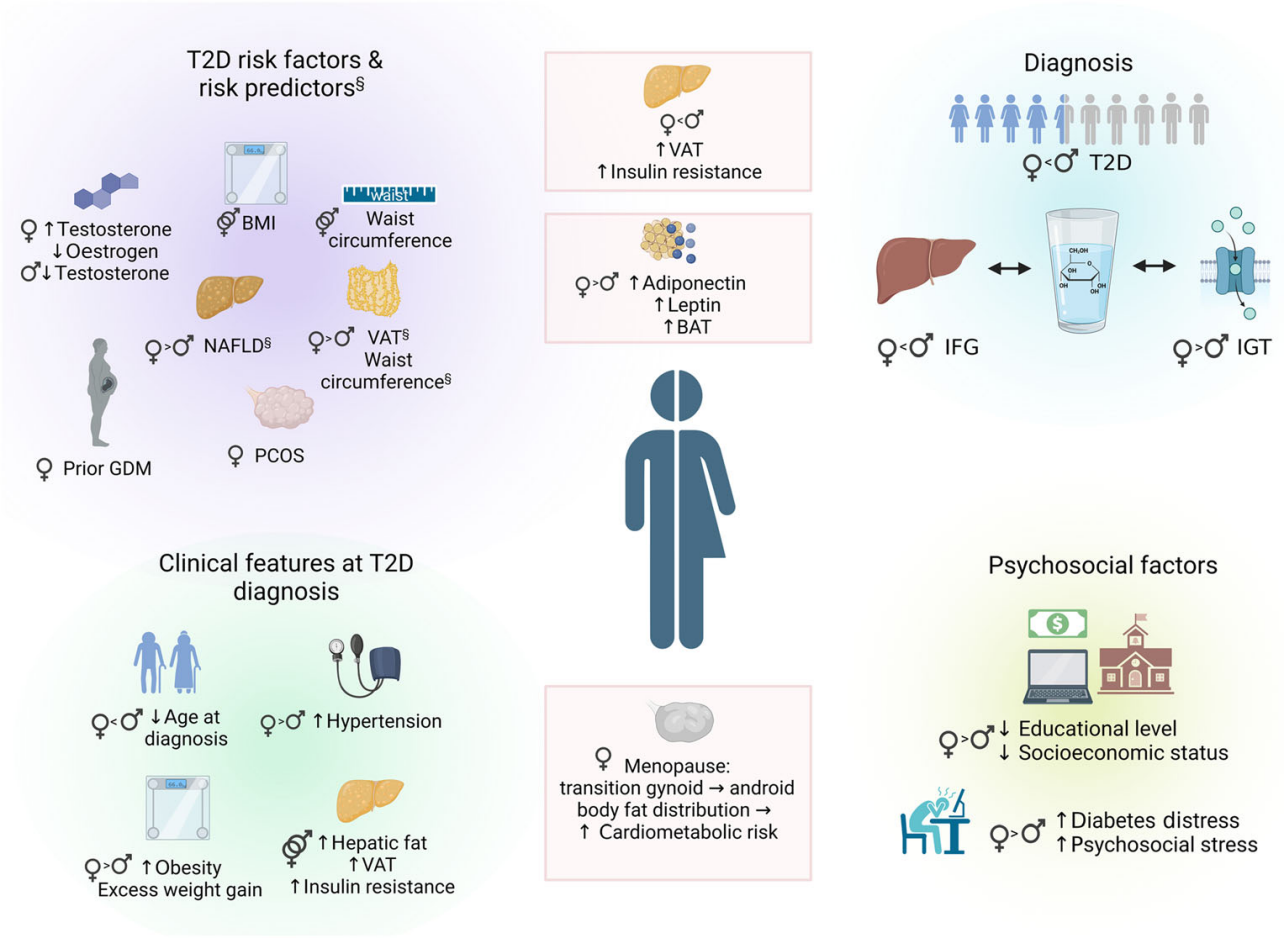
Sex-specific risks and sex and gender differences in risk factors and clinical features of men and women with type 2 diabetes. Significant differences in type 2 diabetes risk predictors between men and women are indicated (§). Physiological characteristics related to type 2 diabetes risk in men and/or women are shown in the centre of the figure in boxes. In general, men have greater insulin resistance and higher fasting glucose levels and higher visceral fat mass than women. However, VAT (or waist circumference as a marker of central obesity) appears to be a better predictor of insulin resistance and development of type 2 diabetes and CVD in women than in men. In women, CVD risk factors like obesity and hypertension progress during menopausal transition, further aggravating insulin resistance, inflammation and dyslipidaemia. At type 2 diabetes diagnosis, women often show larger excess weight gain and higher levels of obesity, as well as higher BP, than men, presenting with an overall higher cardiometabolic disease risk factor burden. Figure created in BioRender.com. This figure is available as part of a downloadable slideset

In general, men develop type 2 diabetes at a younger age and lower BMI [4, 14] (Fig. 1). At the time of type 2 diabetes diagnosis, women often show a higher risk factor burden than men, including higher BP and larger excess weight gain. This particularly applies to white women and younger women [14, 15]. Waist circumference indicates visceral adipose tissue (VAT) more accurately than BMI in women and thus represents a more reliable cardiometabolic risk predictor. This may be ascribed to more prominent loss of muscle and bone mass with increasing age and a greater increase of VAT following menopause in women compared with men of similar age [7, 16]. Indeed, a GWAS confirmed VAT as a stronger independent type 2 diabetes risk factor in women than in men (OR 7.3 vs 2.5) [17].

Both waist circumference and BMI showed significant relationships with mortality among patients with type 2 diabetes [18]. A meta-analysis demonstrated a non-linear association between BMI and mortality in men and women with type 2 diabetes, but mortality risk at higher BMI only increased significantly in women [19]. Diabetes risk scores and mortality prediction models including sex in risk calculations, together with anthropometric measures, hypertension and lipids, can help identify high-risk individuals [4, 14]. Adding novel biomarkers and risk factors like gestational diabetes (GDM) and psychosocial factors may further increase prognosis.

Although younger women show greater levels of adiposity for a given BMI, they are at lower cardiometabolic risk than men of a similar age. The presence of two X-chromosomes has been related to increased adiposity, possibly through enhanced expression of genes involved in weight gain, which escape X-chromosome inactivation [20]. Furthermore, women have a greater ability for adipose tissue expansion in gluteofemoral and subcutaneous fat, conferring better metabolic health [4, 20]. Whereas men tend to be diagnosed with type 2 diabetes at a lower BMI than women, the associations between obesity indices including BMI and type 2 diabetes risk were generally stronger in women than in men [4, 14, 21]. This may be caused by sex-dimorphic body composition and was recently confirmed by Mendelian randomisation analysis (MRA) [22]. However, another MRA showed comparable effects of BMI on type 2 diabetes in both sexes [23]. Moreover, BMI was associated with coronary artery disease in men and premenopausal women, suggesting that excess obesity mitigates the natural protection of young women. Overall, more research is necessary to better clarify the performance of various obesity indicators in the prediction of complications in men and women of different age groups.

Premenopausal women accumulate more gluteofemoral fat (gynoid shape), providing a safe fat reservoir for excess energy and releasing beneficial adipokines, contributing to their higher circulating adiponectin and leptin concentrations [7]. Women also have a greater prevalence of brown adipose tissue (BAT), which affects energy metabolism and is inversely related to age and BMI. Cold-activated BAT and thermogenesis were higher in premenopausal women than in age-matched men and were independently associated with oestradiol levels [24]. BAT was recently demonstrated to be negatively associated with type 2 diabetes and CVD, possibly contributing to women’s lower type 2 diabetes risk [25].

However, post-menopause, fat distribution in women transitions to an android rather than a gynoid pattern, accompanied by an increase in cardiometabolic risk. There are clear sex differences in ectopic fat accumulation that change over a person’s lifetime. In general, healthy women have higher intramyocellular fat in leg muscles but lower VAT, liver and pancreas fat [7, 26], and lower myo- and pericardial lipids than men [4]. However, with deterioration of glucose tolerance these sex differences disappear. Women with prior GDM or polycystic ovary syndrome (PCOS) already show changes in ectopic lipids, which may predict metabolic derangements [27, 28]. In type 2 diabetes, women show liver and pancreas fat levels as high as those in men, related to increased hepatic VLDL1 triacylgycerol production [26]. Moreover, intra-pancreatic fat, which impacts beta cell function, increases with age, especially in women [29].

### Prediabetes

In general, IGT is consistently found to be more common in women than in men, but IFG is diagnosed more often in men (Fig. 1). Higher stimulated glucose values in women may be a consequence of the standard glucose challenge of 75 g OGTTs, if we ignore sex-dependent variables like body size, muscle mass, physical fitness or gastric emptying [4, 14]. Furthermore, prolonged gut glucose absorption may contribute to higher 2 h glucose levels in women compared with men [30]. IFG is mainly caused by increased hepatic insulin resistance and impaired basal insulin secretion, while IGT mainly results from peripheral insulin resistance and reduced stimulated insulin secretion [31]. Furthermore, IFG increases the risk of stroke in men, but IGT increases CHD risk in women [32]. All forms of prediabetes, including definition by HbA_1c_, were related to higher all-cause mortality in both sexes, but composite cardiovascular events were higher in women [33]. MRA suggests that HbA_1c_ may underestimate fasting glucose in men, possibly driven by sex-specific higher iron levels [34]. Thus, we recommend greater use of OGTTs, particularly in women, and measurement of HbA_1c_ in addition to fasting glucose in all individuals. Moreover, higher 1 h post-load glucose levels identified individuals with normal glucose tolerance who are at risk of future type 2 diabetes and CVD [35]. Future studies should clarify whether this value can improve detection of high-risk individuals and reduce gender bias.

### Endocrine factors

Sex steroid hormones largely contribute to sex-dimorphic diabetes susceptibility [4, 14, 36]. In premenopausal women, oestrogen protects from type 2 diabetes by increasing insulin sensitivity and glucose-stimulated insulin secretion, and mitigating beta cell apoptosis. Hence, premature menopause is associated with an increased risk of type 2 diabetes, whereas hormone replacement therapy may prevent or delay type 2 diabetes [36, 37]. One of the most sexually dimorphic metabolic aspects is testosterone’s bidirectional modulation of glucose homeostasis [38]. In men, testosterone physiologically enhances glucose-stimulated insulin secretion, increases GLP-1 action and reduces inflammation, thereby maintaining beta cell health [38]. Interestingly, low levels of free testosterone and high levels of sex hormone binding globulin (SHBG) were independently associated with mortality in men with type 2 diabetes [39]. High SHBG impacts health through the regulation of bioactive testosterone and reduction of tissue androgenisation, and also exerts additional direct effects. However, low SHBG is associated with insulin resistance and type 2 diabetes risk [4], and mediates the association between intrahepatic fat and type 2 diabetes, with a more significant impact in women [40].

Conversely, in women, increased testosterone leads to insulin hypersecretion, mitochondrial dysfunction, oxidative stress and beta cell dysfunction [38]. Thus, testosterone deficiency predisposes men to type 2 diabetes, while androgen excess increases type 2 diabetes risk in women. This is evidenced by an up to fourfold higher risk of glucose alterations in women with PCOS and androgen excess [4, 41]. Interestingly, MRA has revealed that obesity, testosterone and SHBG play a causal role in PCOS, but PCOS had no direct causal effect on type 2 diabetes or CVD [42].

In contrast, a double-blind RCT in which overweight men, aged 50–74 with low testosterone and IGT or newly diagnosed type 2 diabetes, were enrolled in a lifestyle programme showed that intramuscular testosterone therapy could prevent or revert type 2 diabetes by 41% within 2 years of treatment, compared with placebo [43]. Thus, screening for hypogonadism should be considered in men with type 2 diabetes and obesity. Possible benefits and risks of testosterone in addition to behavioural or glucose-lowering therapy should be discussed with men who have a testosterone deficiency.

### Pregnancy

Pregnancy may unmask subtle pre-existing metabolic disturbances, leading to a high percentage of women developing GDM (5–16%) [4]. GDM is a heterogeneous entity mostly affecting insulin-resistant women with obesity, but lean women with reduced beta cell capacity, who are therefore less able to compensate for pregnancy-related insulin resistance, can also be affected [44]. GDM is diagnosed more frequently in older women and in specific ethnic groups, although there can be high variability in diagnosis due to differences in screening procedures, genetic background, body composition, weight gain or cultural practices [45].

GDM is the most prominent independent risk factor for type 2 diabetes progression in women [46]. A recent meta-analysis showed that women with GDM had a relative risk (RR) of type 2 diabetes of 8.3 (95% CI 6.5, 10.6). The percentage of type 2 diabetes diagnoses was 12% higher for each year following pregnancy, 18% higher per BMI unit at follow-up and 57% lower in White European women than in women from other populations [47].

Although intervention strategies are an effective approach to reducing incident type 2 diabetes, in the Diabetes Prevention Program, the incidence of type 2 diabetes in women with prior GDM was still 70% higher over 3 years than in women with prediabetes or normoglycaemia in previous pregnancies [48]. Therefore, sustained glucose monitoring over time and implementation of suitable prevention programmes is recommended in high-risk women with prior GDM.

The global rise in adiposity may explain the huge increase in pregestational type 2 diabetes, the most common form of pregestational diabetes in many countries today [49]. The largest study of such pregnancies showed low rates of contraception use, inadequate preconceptual care, insufficient glycaemic control during pregnancy, high rates of comorbidities, and pregnancy-related complications [50]. One in four of these women experienced intrauterine death. Therefore, better and more personalised preconception and antenatal care is particularly important for young women with early-onset type 2 diabetes.

### Psychosocial factors

A low level of education, low socioeconomic and occupational status, and low income are all significant risk factors for the development of type 2 diabetes, especially in women [4, 51, 52] (Fig. 1). Higher household income has stronger effects on type 2 diabetes risk and is positively related to the prevalence rate in men in developed countries [53], but the effect of income is complex and varies globally, and also depends on a country’s Human Development Index (HDI) [4]. Additionally, access to healthcare, particularly in women from developing countries, can be a barrier for sufficient prevention and treatment of type 2 diabetes [54]. In Japan, higher levels of perceived stress were closely related to an increased risk of incident diabetes, with stronger effects in men [55]. High work-demands and an active job in general seem to be stronger protective factors in men, while low decision latitude shows stronger associations with type 2 diabetes development in women [4, 56]. Sedentary time is closely related to anxiety, depressive symptoms, higher perceived interference and lower self-efficacy, with stronger effects in women with type 2 diabetes [57]. Prolonged night work was related to an increased type 2 diabetes risk only in women (HR 1.46) [58]. Similar results for shift work have been reported in women [59]. In conclusion, psychosocial risk factors have a stronger impact on the development of type 2 diabetes in women compared with men. To reduce or prevent type 2 diabetes risk, especially in women, it would be necessary to screen patients with metabolic disorders that are closely related to the development of type 2 diabetes (e.g. obesity or prediabetes) for psychosocial risk factors at an early stage.

**Fig. 2:**
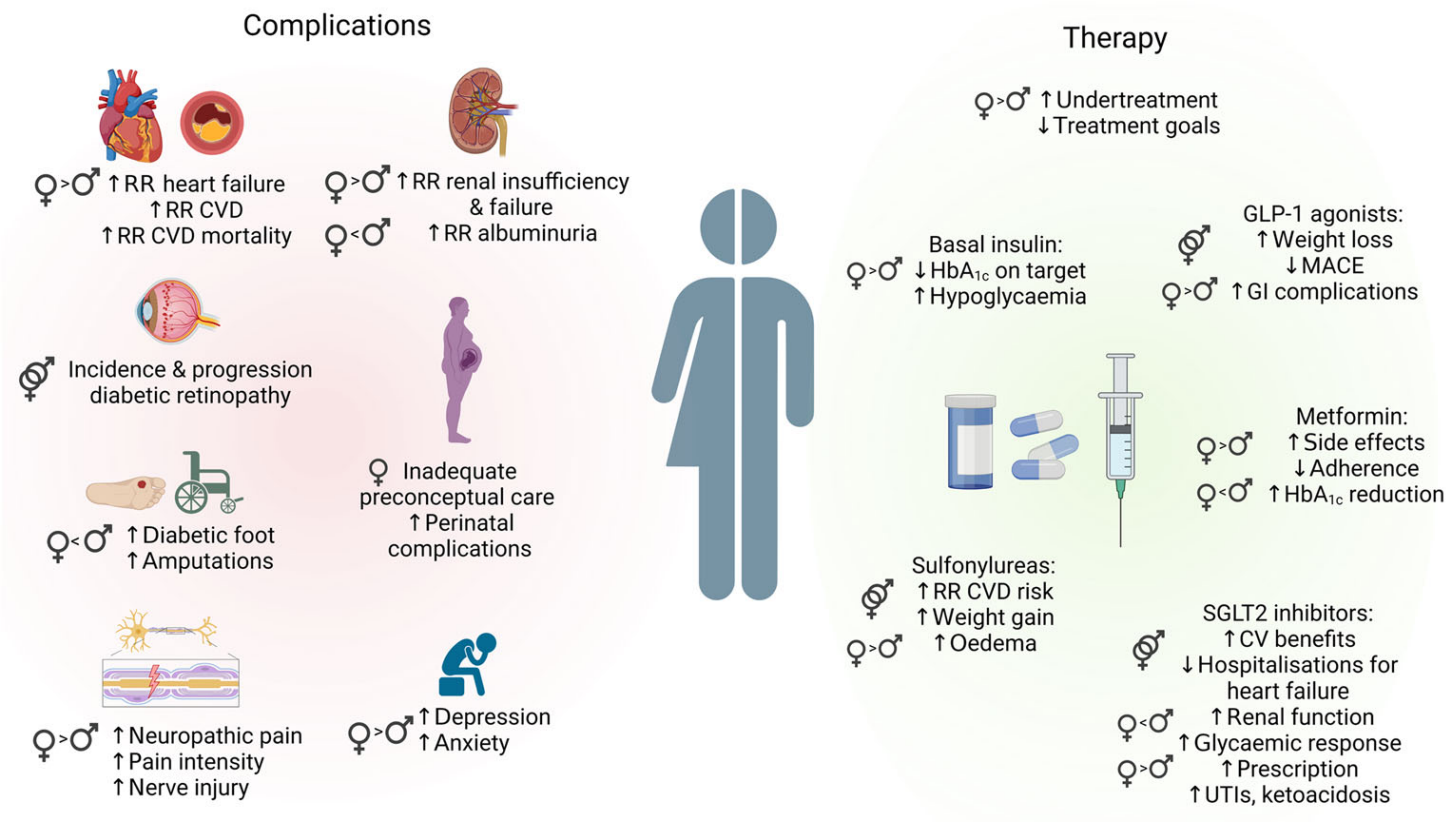
Illustration of the most important sex differences in the complications and possible effects of pharmacological therapy and management of patients with type 2 diabetes. CV, cardiovascular; GI, gastrointestinal; HF, heart failure; UTI, urinary tract infection. Figure created in BioRender.com. This figure is available as part of a downloadable slideset

Sex and gender differences were evidenced in type 2 diabetes-related comorbidities such as CVD and cancers but also psychiatric disorders, including anxiety and depression (Fig. 2) [4, 60, 61]. There is a higher prevalence of depression in women than men, and this is particularly seen when women reach menopause [61]. Higher rates of depression and less problem-oriented and -solving activities in women with type 2 diabetes may ultimately lead to reduced self-care activities [62]. Psychiatric disorders like depression increase the probability of an unhealthy lifestyle and reduce adherence to therapeutic recommendations. Overall, prevalence of diabetes distress is very common among patients with type 2 diabetes (~36%) and is clearly associated with comorbid depressive symptoms, anxiety and female gender [63]. It is important to recognise the large overlap between diabetes distress and depression in order to enable appropriate screening and patient-centred care, possibly improving medication adherence and outcomes among patients with type 2 diabetes.

### Macrovascular complications

In men and women, CVD is the leading cause of death. Type 2 diabetes contributes to premature mortality from CVD, with some variations resulting from sex differences (Table 1) [4, 44]. Although the absolute risk of CVD mortality is higher in men with type 2 diabetes, the RR is significantly greater in women with type 2 diabetes [44, 64, 65], although a separate study showed it comparable between sexes [66]. A recent MRA showed evidence of causal effects of type 2 diabetes on CHD risk but without sex dimorphism [67]. Interestingly, higher relative mortality risks in individuals with type 2 diabetes were found at younger ages; 35–59 year old women were the most affected group [65]. One potential reason was that women with type 2 diabetes are more likely to have advanced atherosclerosis than men at the time of diagnosis [5]. In young women, the development of type 2 diabetes is associated with greater weight gain, which subsequently leads to a more adverse cardiometabolic risk profile [44]. Even with mild dysglycaemia, women present with subclinical inflammation and increased coagulopathy from early adulthood onwards [44]. These aspects could explain why the highest RR for CVD was recently reported in younger women with type 2 diabetes [65, 68]. For women, a higher RR of cardiovascular-related death associated with newly diagnosed type 2 diabetes has been described previously, particularly among individuals who smoke, have hypertension or hypercholesterolaemia, or are overweight [69]. Therefore, it was assumed that hyperglycaemia has stronger synergistic effects on these risk factors in women than men, and that more aggressive intervention is needed in women to curb cardiovascular mortality. CVD risk factors, like obesity and hypertension, progress during menopausal transition, further aggravating insulin resistance, inflammation and dyslipidaemia in women with type 2 diabetes [66].

**Table 1:** Sex and gender differences in macro- and microvascular complications in men and women with type 2 diabetes mellitus listed trait-specific (adapted from [4, 44])

Complication	Population	Author	Follow-up duration	Design	Main results
Macrovascular					
Cardiovascular	2,314,292 men and women, T2D, 254,038 all-cause deaths	Xu et al (2019) [84]	Diabetes duration: 5–30 years	Systematic review, 35 prospective cohort studies. Studies from Europe, Asia, Northern America, Australasia	All-cause and CHD mortality: higher pooled risk in women vs men, HR 1.17 (1.12, 1.23) and 1.97 (1.49, 2.61). All-cause mortality: men HR 1.91 (1.72, 2.12), women HR 2.33 (2.02, 2.69) with type 2 diabetes vs healthy population
	5,162,654 men and women, T2D, average baseline age 33.3–80.0 years	Wang et al (2019) [85]	Diabetes duration: 4.7–30 years	Systematic review, 49 studies with 86 prospective cohorts. Studies from Europe, Asia, Northern America, Australasia	All-cause mortality and CVD mortality: higher risk in women vs men with type 2 diabetes, RRR 1.13 (1.07, 1.19) and 1.30 (1.13, 1.49). CHD mortality and stroke mortality: women vs men with type 2 diabetes, RRR 1.58 (1.32, 1.90) and 1.08 (1.01, 1.15)
	447,064 men and women, T2D, age range 15–98 years	Huxley et al (2006) [64]	Diabetes duration: 4—36 years	Meta-analysis, 37 prospective cohort studies. Studies from Europe, Asia, Northern America, Australasia	Fatal CHD: higher risk in women vs men with type 2 diabetes, pooled ratio of the RR 1.46 (1.14, 1.88). Women with diabetes vs no diabetes: 3.50 (2.70, 4.53), men with diabetes vs no diabetes 2.06 (1.81, 2.34)
	980,793 men and women, T2D, 76,965 fatalities, age 35–89 years	Prospective Studies Collaboration and Asia Pacific Cohort Studies Collaboration (2018) [65]	–	Meta-analysis, 68 prospective studies. Studies from Europe, Asia, Northern America, Australasia	Occlusive vascular mortality risk: lower risk in men with type 2 diabetes vs women with type 2 diabetes, RR 2.10 (1.97, 2.24) and 3.00 (2.71, 3.33); younger groups with type 2 diabetes age 35–59 vs older groups age 70–89 years, RR 2.60 (2.30, 2.94) and 2.01 (1.85, 2.19); highest risk: women with type 2 diabetes age 35–59 years RR 5.55 (4.15, 7.41)
	79,985 men and women with T2D matched to 386,547 men and women without T2D, age 60.8–63.9 years	Wright et al (2019) [66]	Follow-up: 3.6±2.4 years	Retrospective cohort study, England, different ethnicities	CVD events: higher risk in both men and women with type 2 diabetes vs healthy population, non-significant higher RR in women vs men RR 1.07 (0.98, 1.17)
	46,606 men and women in trials examining effects of diabetes medications on MACE, age ≥18 years, T2D	Clemens et al (2020) [70]	–	Meta-analysis of 5 CVOTs on 3- or 4-point MACE^a^	Stroke: higher risk in women vs men, RR 1.28 (1.09, 1.50). Heart failure: higher risk in women vs men, RR 1.30 (1.21, 1.40). CKD: higher risk in women vs men, RR 1.33 (1.17, 1.51). PAD: similar risk in women vs men, RR 1.12 (0.97, 1.30). Myocardial infarction: similar risk in women vs men, RR 0.71 (0.59, 0.86). Consistently fewer female participants (28.5–35.8%) in trials
Diabetic foot disease	322,140 men and women, mostly T2D	Seghieri et al (2022) [86]	–	Retrospective study, follow-up 2011–2018, Tuscany, Italy	Diabetic foot disease: higher incidence rate in men vs women, IR 1.57 (1.54, 1.61) and 0.97 (0.94, 1.00)/100,000 PY. Increased risk of adverse events in women when diabetic foot disease associated with vascular pathogenesis
	33,686,171 men and women with diabetes, mostly T2D, ≥18 years	Fan and Wu (2021) [87]	Diabetes duration: >5–17.2 years	Systematic review. Studies from Asia, America, Europe, Australia, Africa	Amputation: higher risk in men vs women, OR 1.38 (1.13, 1.70)
	6,117,981 men and women, T2D, 14,627 lower limb amputations, mean age: 56.5 ±12.7 years	Gandhi et al (2020) [88]	–	Retrospective cohort study, database analysis, 2007–2018, USA	Lower limb amputation: higher incidence rate in men vs women per 1000 PY, IR 1.24 (1.22, 1.26) and 0.46 (0.45, 0.48)
Heart failure	218,549 (46% women) men and women, T2D, age 40–89 years	Malmborg et al (2020) [68]	Diabetes duration: 5–15 years	Population-based study, Denmark	MACE-HF: higher RR in women vs men, RRR 1.15 (1.11, 1.19) at the age of 50–60 years. Higher absolute risk of MACE-HF in men
	12,142,998 men and women, T2D, 253,260 HF events, 90–95%	Ohkuma et al (2019) [73]	Diabetes duration: ≥16 years	Systematic review 47 cohorts. Studies from Europe, Asia, Northern America, Australasia	HF: higher risk in women vs men with type 2 diabetes, RRR 1.09 (1.05, 1.13)
	7785 men and women, T2D, mean age 67.6±10.7 years	Fujita et al (2022) [74]	Median 8 years (IQR 2–16)	Retrospective registry study follow-up 1328 days, Japan	Hospitalisation for HF: higher risk in women vs men with type 2 diabetes and CAD, HR 1.26 (1.06, 1.50)
Microvascular					
Nephropathy	3410 patients; 49% women, 29% T2D, age 40–75 years	de Ritter et al (2021) [79]	Mean diabetes duration: 4–5 years	The Maastricht Study, population-based, observational cohort study, the Netherlands	Sensory nephropathy: higher risk in men with type 2 diabetes vs men with NGT, OR 2.46 (1.67, 3.63). Nephropathy: higher risk in men with type 2 diabetes vs men with NGT, OR 1.58 (1.01, 2.46). Arteriolar diameters: worse parameters in men with type 2 diabetes vs men with NGT, difference: 4.29 μm (1.22, 7.36). Retinal arteriolar dilatation: worse parameters in men with type 2 diabetes vs men with NGT, difference: −0.74% (–1.22, 0.25)
	1470 men and women, T2D, age 65±11 years	de Hauteclocque (2014) [89]	Median follow-up 5.7 years, France	Prospective cohort study, France	eGFR: women −1.31 ml^−1^ min 1.73 m^−2^ per year, men −1.77 ml min^−1^ 1.73 m^−2^ per year. ESRD: higher risk in men vs women
	344 patients (247 male, 97 female), T2D, age <65 years	Kajiwara et al (2016) [90]	Follow-up duration: 8.1±1.4 years. Diabetes treatment <5 years	Retrospective longitudinal study, Japan	eGFR: women −3.5±2.7% per year, men −2.0±2.2% per year. HbA_1c_ and LDL-cholesterol levels significantly associated with eGFR decline in women
	5102 men and women, T2D, age 25–65 years	Retnakaran et al (2006) [81]	Median follow-up time 15 years	UK Prospective Diabetes Study (UKPDS), UK, Caucasian, Indian Asian, Afro-Caribbean populations	Microalbuminuria: Higher risk in men vs women, HR 1.18 (1.00, 1.39). Macroalbuminuria: higher risk in men vs women, HR 1.42 (1.05, 1.49). eGFR <60 ml/min per 1.73m^2^: lower risk in men vs women, HR 0.43 (0.48, 0.59)
Neuropathy	Cohort 1: 223, cohort 2: 128 men and women, T2D, age ≥18 years	Abraham et al (2018) [82]	Diabetes duration cohort 1: 11–12 years, cohort 2: 13–15 years	Cohort 1: prospective, cohort 2: retrospective, Canada	Neuropathic pain: higher risk in women vs men, 68% and 53%. Pain intensity: greater VAS in women vs men, 7.9–8.5 and 6.8–6.9
	376 men and women (59% women) with T2D, age ≥18 years	Aaberg et al (2008) [91]	–	Retrospective chart analysis, USA, African, Caucasian and Asian populations	Neuropathic complications: earlier onset in men vs women, 63 vs 67 years
Retinopathy	214 patients (119 men and 95 women), T2D, mean age 63±12	Nakayama et al (2021) [83]	Median diabetes duration: 10 years	Retrospective analysis, Japan	Retinopathy: no significant sex differences in incidence or progression
	383 men and women, T2D, mean age 59.4±11.0 years	Kajiwara et al (2014) [92]	Follow-up 5.8 years, diabetes duration: 11.0± 8.3 years	Retrospective longitudinal study, Japan	Proliferative diabetic retinopathy: higher prevalence in women vs men, female sex is independent risk factor

Data are presented with 95% CI in parentheses, unless otherwise stated

^a^3- or 4-point MACE: Cardiovascular death, non-fatal myocardial infarction, non-fatal stroke, hospitalisation for heart failure, hospitalisation for unstable angina for 4-point MACE

CAD, coronary artery disease; CKD, chronic kidney disease; CVOT, cardiovascular outcome trial; ESRD, end-stage renal disease; HF, heart failure; IR, incidence rate; NGT, normal glucose tolerance; PAD, peripheral arterial disease; PY, patient years; RRR, women-to-men ratio of RRs; T2D, type 2 diabetes; VAS, visual analogue scale

Additionally, medication adherence or prescriptions treating several CVD risk factors were lower in women than men with type 2 diabetes. In cardiovascular outcome trials, less use of statins, aspirin and beta blockers in women with type 2 diabetes was reported, despite the higher prevalence of history of stroke and heart failure [70]. Accordingly, women had higher BP, LDL-cholesterol and glucose variables than men. Therefore, regardless of their comorbidities, fewer women with type 2 diabetes were treated in accordance with the guidelines than men [70]. Similarly, in a recent EUROASPIRE survey, women with type 2 diabetes or IGT were older and less likely to meet the recommended targets for physical activity, BP or LDL-cholesterol than men, probably contributing to their higher CVD risk [71]. Furthermore, a Danish cohort study reported that cardioprotective glucose-lowering drugs, such as sodium–glucose cotransporter 2 inhibitors (SGLT-2I) or GLP-1 receptor agonists (GLP-1RA), are prescribed less often for women with type 2 diabetes and CVD [72]. Health professionals thus appear to underestimate CVD risk in women with type 2 diabetes [44]. Ultimately, this leads to less use of CVD protective medication and inadequate CVD risk factor management, which needs to start as early as possible.

Among patients with type 2 diabetes, women also have a greater RR of heart failure and hospitalisation due to heart failure than men [73, 74]. This was also evidenced at a younger age, although the women-to-men ratio fell with increasing age [68]. Hypertension is a main driver of heart failure progression, especially in women with hypertension compared with women without hypertension (threefold increase in risk in for women vs twofold for men) [75]. Sex-specific analyses demonstrated faster progress of BP elevation in young women, starting as early as the third decade [76]. Type 2 diabetes has a more pronounced effect on heart failure progression in women (women: 5-fold vs men: 2.4-fold risk) [77]. Women suffer more often from diastolic dysfunction caused by hypertension, insulin resistance and obesity, and thus more frequently develop heart failure with preserved ejection fraction (HFpEF) [78].

### Microvascular complications

Evidence of sex differences in microvascular disease is scarce and inconclusive (Table 1). Men with type 2 diabetes showed a higher risk of sensory neuropathy, nephropathy and worse retinal microvascular measures than men with normoglycaemia, while this was not evident among women [79]. Nonetheless, among patients with type 2 diabetes, a higher risk of renal failure and renal insufficiency was observed in women, possibly due to less intensive risk factor therapy, although higher risk of albuminuria was found in men [80, 81]. Women with type 2 diabetes reported more frequent and greater neuropathic pain and nerve injury than men [82]. No sex differences in diabetic retinopathy were recently observed [83]. Further research in this area is thus urgently needed.

## Sex differences in pharmacological therapy and management

The scarce literature about lifestyle interventions on cardiometabolic health in humans suggests that, under lifestyle interventions, men have greater success with weight and body fat reduction, with a greater general cardiometabolic benefit, than women [4, 93]. In the DiRECT weight management programme, type 2 diabetes remission was also more durable in men at 2 years, probably due to greater weight loss [94].

Sex differences in the pharmacological management of type 2 diabetes and the response to treatment (Table 2 and Fig. 2) demonstrate that undertreatment is a major problem in women [95]. Metformin is one of the most-prescribed glucose-lowering drugs and evidence suggests that women are less adherent to this therapy and more likely to suffer side effects [96, 97]. Despite comparable bioavailability, a greater HbA_1c_ reduction in men has been shown [98]. Moreover, both metformin therapy and lifestyle intervention in women with prior GDM showed a strong protective effect regarding type 2 diabetes progression [99].

**Table 2:** Potential sex and gender differences in (adverse) effects of glucose-lowering- and cardiovascular medications in patients with type 2 diabetes

Therapy	Efficacy and adverse effects	Notes
Metformin	Glucose metabolism	Men: greater HbA_1c_ reduction. Women: greater reduction of body weight [98]
	Adherence	Women: less adherent to therapy [96]
	Adverse effects	Women: more likely to suffer side effects (e.g. gastrointestinal symptoms) [96]; increased hospitalisation rate [97]
	Possible fetal programming effects	Mother undergoing metformin therapy: higher rates of infants born SGA and of childhood adiposity, possibly with slightly higher risk in boys [121]; father undergoing metformin therapy: exposure associated with major birth defects, particularly genital birth defects in boys [122]
Sulfonylureas	Glucose metabolism	Male sex and lower BMI: greater HbA_1c_ reduction, similar hypoglycaemia risk [100]
	Outcome	Women and men: higher risk of CHD [101, 102]
Thiazolidinediones	Glucose metabolism	Women with obesity: greater HbA_1c_ reduction compared with sulfonylurea therapy [100]
	Adverse effects	Women: women with obesity have a higher risk of weight gain and oedema risk [100]; higher risk of bone fractures; women with type 2 diabetes have a higher mortality rate under a therapy with rosiglitazone [62]. Men: pioglitazone is related to a moderately increased risk of bladder cancer [123]
SGLT-2I	Glucose metabolism	Men: (trend for) better glycaemic response to treatment [124]
	Outcome	Similar between men and women: cardiovascular benefits, risk of hospitalisation due to heart failure and changes (incident or worsening) in nephropathy [102, 104, 105]. Women: lower prescription rate [103]
	Adverse effects	Women: higher risk of adverse events in general, higher risk of genital infection or urinary tract infections, more urosepsis, fractures and ketoacidosis [102, 108, 109]. Men: higher risk of Fournier gangrene, more acute renal failure, more lower limb amputation, more pancreatitis [102, 125]. Similar between men and women: risk of adverse events in general, amputation and genital infection or urinary tract infections [104]
GLP-1RA	Weight reduction	Women: greater weight reduction [110–113]
	Glucose metabolism	Majority of the trials: similar HbA_1c_ reduction between sexes [110, 112]. Men: better glycaemic control under add-on exenatide therapy (to metformin ± sulfonylurea). Women: combination therapy with exenatide and metformin is more effective [113]. Dulaglutide: similar HbA_1c_ reduction in men and women [112]. General: female sex could be a predictor of better glycaemic response [62, 126]
	Outcome	Similar reduction of MACE in men and women [114, 115]. Women: comparison GLP-1RA vs sulfonylurea: better CV-reducing effect [102]
	Adverse effects	Women: greater risk of gastrointestinal complications (e.g. nausea + diarrhoea) [62, 112, 127]
DPP-IV inhibitors	Glucose metabolism	No sex differences [116]
Insulin	Glucose metabolism	Women: achieve HbA_1c_ targets (<7%) with basal insulin glargine less often [117]
	Adverse effects	Women: higher risk of severe (nocturnal) hypoglycaemic events compared with men with basal insulin therapy with NPH insulin or insulin glargine, especially if without obesity [128]. Men: in a Japanese study in which patients with a mean BMI of <25 kg/m^2^ with longstanding type 2 diabetes received a CSII for 7 days and subsequent therapy with premixed insulin, men had a higher risk of hypoglycaemia, although they required lower doses of insulin; similar results have been shown for CSII therapy only [118]
Statins	Cardiovascular outcomes	Similar effects in men and women undergoing statin therapy [129]
	Volume reduction of coronary atheroma	Women: stronger reduction in women undergoing high-dose statin therapy [130]
Evolocumab	Coronary atheroma reduction	Women: more relative (but not total volume) coronary atheroma reduction [131]
Fenofibrate	Lipid-lowering effect and outcome	Women: more total, LDL- and non-HDL-cholesterol reduction compared with men, similar CVD outcomes [132]
ACE inhibitors	Outcome	Women: decreasing efficacy over time, less reduction of mortality rate but greater beneficial effects on nephropathy [133]
	Adverse effects	Women: experience side effects (cough) more often than men [134]
ACE inhibitors and ATII blockers	HFrEF	Comparable in both sexes: mortality and hospitalisation due to HFrEF [134]
Beta blockers	Outcome	Women: optimal survival under lower dosages of beta blockers [5]
Acetylsalicylic acid	Outcome	Women: no reduction of MCI risk, but reduction of ischaemic strokes, increased bleeding risk [135]. Men: MACE reduction exclusively in men [136]
ARNI	HFpEF	Women: decreased risk of cardiovascular death or hospitalisation with HF [137]. Specific subgroup analysis: only in women reduction of the primary endpoint cardiovascular death and hospitalisation due to HF [138]

ARNI, angiotensin receptor–neprilysin inhibitor; ATII, angiotensin II; CSII, continuous subcutaneous insulin infusion; CV, cardiovascular; DPP-IV, dipeptidyl peptidase-4; GLP-1RA, glucagon-like peptide-1 receptor agonist, HF= heart failure; HFrEF, heart failure with reduced ejection fraction; MCI, myocardial infarction; SGA, small for gestational age

Body composition and BMI play an important role in the sex-specific glycaemic response to sulfonylurea therapy [5, 100]. However, sulfonylureas were related to an increased risk of CHD in both sexes [101, 102]. In a study of people taking thiazolidinediones, adverse drug reactions such as weight gain, risk of oedema and risk of bone fracture predominated in women. Its usage should thus be limited in women, especially after menopause [100].

Regarding the improvement of glucose metabolism, there is slight evidence that the response to SGLT-2I treatment is better in men. Although therapy with SGLT-2I dramatically reduces the risk of CVD and heart failure, and improves renal function, SGLT-2Is are more frequently prescribed to men [72, 103]. A gender-pooled meta-analysis revealed that the effects of SGLT-2Is on major adverse cardiovascular events (MACE), hospitalisation for heart failure, cardiovascular death, and fatal or non-fatal stroke or myocardial infarction were comparable between men and women [104]. In another trial, empagliflozin reduced the risk of cardiovascular-related death and heart failure-related hospitalisation to a comparable degree, with similar health benefits in men and women with HFpEF, both with and without type 2 diabetes, regardless of their baseline ejection fraction [105]. A sex-stratified subgroup analysis confirmed these results, including the comparable benefits between men and women, in patients treated with dapagliflozin, who had mildly reduced heart failure or HFpEF. This was probably because of the large number of women included and a predominance of women in the group with the highest ejection fraction [106].

Although there has previously been evidence showing a higher prevalence of genital and urinary tract infections in women undergoing SGLT-2I therapy [107], more recent analysis did not report sex differences for vascular efficacy, amputation, fracture risk, genital infection or urinary tract infections [104]. However, ketoacidosis [108] and an increased fracture risk with canagliflozin has been reported for women [109].

GLP-1RA also show significant sex differences and cumulative evidence suggests that women display greater weight reduction [62, 110–113]. The majority of the clinical trials report similar HbA_1c_ reduction with different GLP-1RAs in both sexes [110, 112]; however, a combination therapy of exenatide and metformin appeared to be especially effective in women [113]. Although no sex differences are reported for MACE [114, 115], women have greater risk of gastrointestinal side effects with GLP-1RAs [62]. For gliptins there is no evidence of sex differences in HbA_1c_ reduction [116].

A meta-analysis showed that women less frequently met the HbA_1c_ target of <7% with basal insulin therapy, with insulin glargine, or with NPH insulin, despite a higher risk of severe hypoglycaemic events [117]. However, in Asian patients with longstanding type 2 diabetes, a therapy with premixed insulin following continuous subcutaneous insulin infusion (CSII) therapy was related to a higher risk of hypoglycaemia in normal-weight men [118]. Thus, insulin management may need special attention in women and normal-weight individuals, although further research is necessary.

There are also significant sex differences in lipid-lowering drugs, ACE inhibitors, angiotensin II (ATII) blockers, aspirin and angiotensin receptor–neprilysin inhibitors (ARNI), which are commonly prescribed medications in patients with type 2 diabetes. Statins appear to offset increased cancer risk, which is otherwise commonly seen in patients with diabetes, independent of age and sex [60]. However, dose-dependent effects were described, with higher rates of osteoporosis and depression, especially in postmenopausal women on high doses [119, 120].

## Future perspective

More research into sex and gender differences in type 2 diabetes is essential for a better understanding of the biological background and psychosocial impact. Sex and gender differences are interdependent on age or ethnicity and disentangling these connections will allow further personalisation of diabetes management. Increased alertness in specific subgroups like ethnic minorities, and particularly young patients with type 2 diabetes if their glycaemic variables frequently fall outside of the target range, is essential. These high-risk groups which develop type 2 diabetes at a lower BMI and younger age need accurate screening and special targeted prevention. Earlier detection of type 2 diabetes and concomitant cardiovascular risk factors is crucial to prevent CVD events. Additionally, weight management appears to be essential in type 2 diabetes prevention and therapy in women, in combination with the additional use of effective new drugs with cardiorenal benefits and individualised lifestyle intervention approaches. More attention should also be paid to BP management, especially in women with obesity and type 2 diabetes, who display a higher cardiovascular risk at a young age. As women with type 2 diabetes have a higher RR of CVD, clinicians need to focus on more intense treatment of risk factors to reduce vascular comorbidities. Lower treatment thresholds in women might help to lower CVD but require evidence from clinical studies and follow-up. At present, aggressive multifactorial treatment in accordance with current guidelines is essential and needs to be delivered to all people with type 2 diabetes, independent of sex, age or ethnicity. Depression and diabetes distress are essential factors undermining diabetes management and self-care activities, particularly in women. Higher awareness, better screening tools, psychological support and research are needed to help overcome these gender gaps. Furthermore, educational approaches for physicians and the public may help to further increase awareness of type 2 diabetes and its sequelae in men and women.

## Conclusions

Sex and gender differences in type 2 diabetes encompass biological and psychosocial risk factors, pathophysiology and complications, but also its treatment and adherence to it, mostly demonstrating a higher RR of cardiovascular diabetes complications in women with type 2 diabetes (see text box). This is most obvious for macrovascular complications in women, who have an increased RR of CVD mortality, possibly driven by risk factor burden and loss of natural protection after menopause. A special focus in research on and clinical routine for vulnerable groups such as women with prior GDM or men and women with reproductive disorders and obesity is needed. These groups could potentially benefit from targeted prevention programmes and more intense, sex-specific risk reduction approaches. However, targeted treatment strategies in type 2 diabetes require further investigation in future trials.

## Supplementary information

ESM 1 (PPTX 4.21 mb)

## Abbreviations

BAT: Brown adipose tissue
GDM: Gestational diabetes mellitus
GLP-1: Glucagon-like peptide-1
GLP-1RA: Glucagon-like peptide-1 receptor agonist
HFpEF: Heart failure with preserved ejection fraction
IFG: Impaired fasting glucose
IGT: Impaired glucose tolerance
MACE: Major adverse cardiovascular events
MRA: Mendelian randomisation analysis
NAFLD: Non-alcoholic fatty liver disease
PCOS: Polycystic ovary syndrome
RR: Relative risk
SGLT–2I: Sodium–glucose transport protein 2 inhibitor
SHBG: Sex hormone binding globulin
VAT: Visceral adipose tissue
