# Correction to: Sex differences in type 2 diabetes

**DOI:** 10.1007/s00125-023-05913-8

**Published:** 2023-04-12

**Authors:** Alexandra Kautzky-Willer, Michael Leutner, Jürgen Harreiter

**Affiliations:** 1 Department of Medicine III, Division of Endocrinology and Metabolism, Medical University of Vienna, Vienna, Austria; 2 Gender Institute, Lapura Women’s Health Resort, Gars am Kamp, Austria

**Correction to: Diabetologia**

**https://doi.org/10.1007/s00125-023-05891-x**

The original text mistakenly stated that premenopausal women have higher skeletal muscle mass than men. The original text has now been corrected to read: ‘Studies have provided evidence that premenopausal women have higher skeletal muscle and hepatic insulin sensitivity and higher stimulated insulin secretion, and thus lower fasting glucose and HbA_1c_ values, than men [4, 7]’.

