# Supplementary material for: Sex differences in type 2 diabetes
Source: Diabetologia. 2023 Mar 10;66(6):986–1002. doi: 10.1007/s00125-023-05891-x (PMC10163139; doi:10.1007/s00125-023-05891-x)
Supplement: Supplementary file 1 — (PPTX 4.21 mb) [file 125_2023_5891_MOESM1_ESM.pptx]

## Slide 1
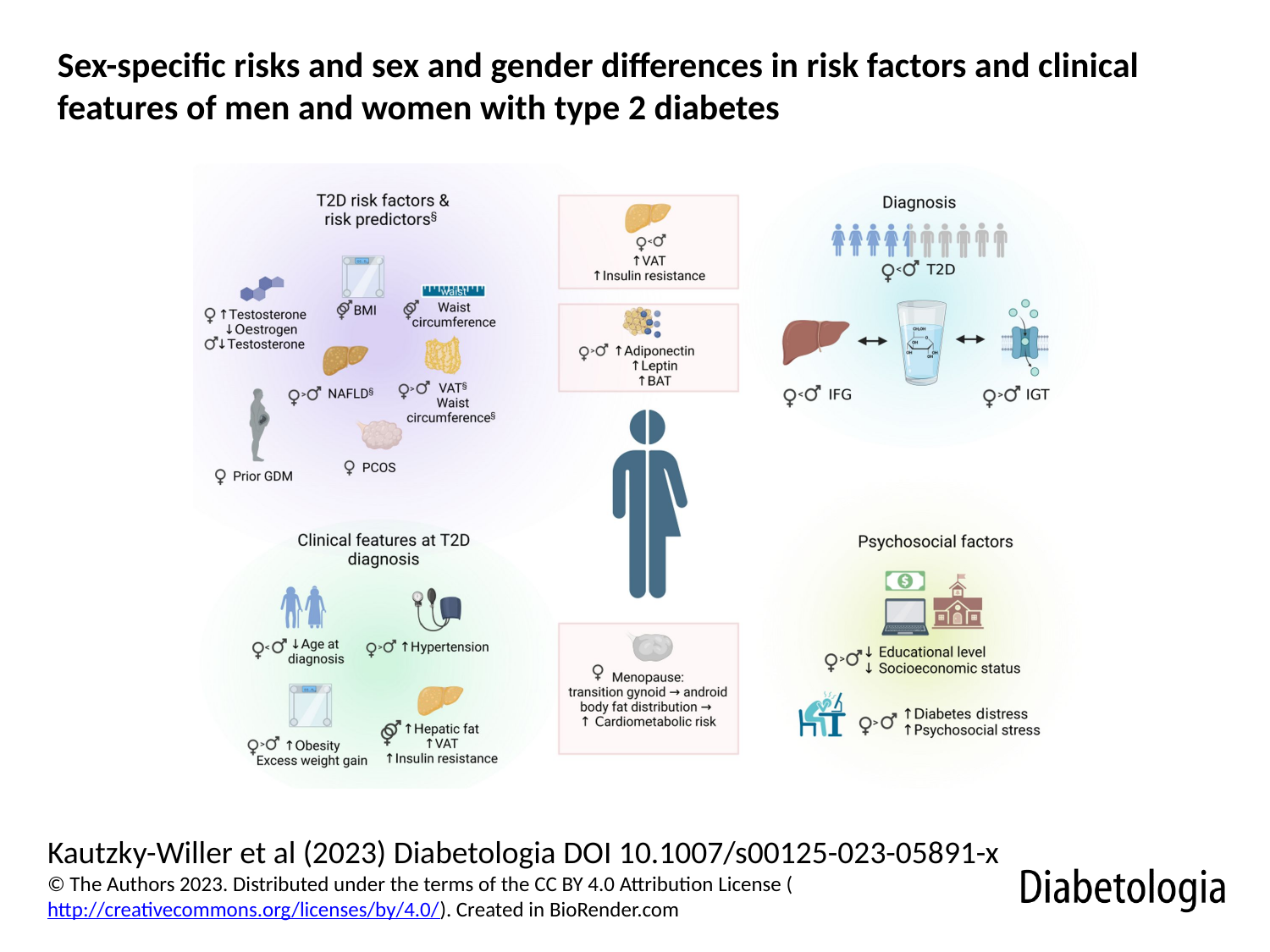

Sex-specific risks and sex and gender differences in risk factors and clinical features of men and women with type 2 diabetes
Kautzky-Willer et al (2023) Diabetologia DOI 10.1007/s00125-023-05891-x
© The Authors 2023. Distributed under the terms of the CC BY 4.0 Attribution License (http://creativecommons.org/licenses/by/4.0/). Created in BioRender.com

## Slide 2
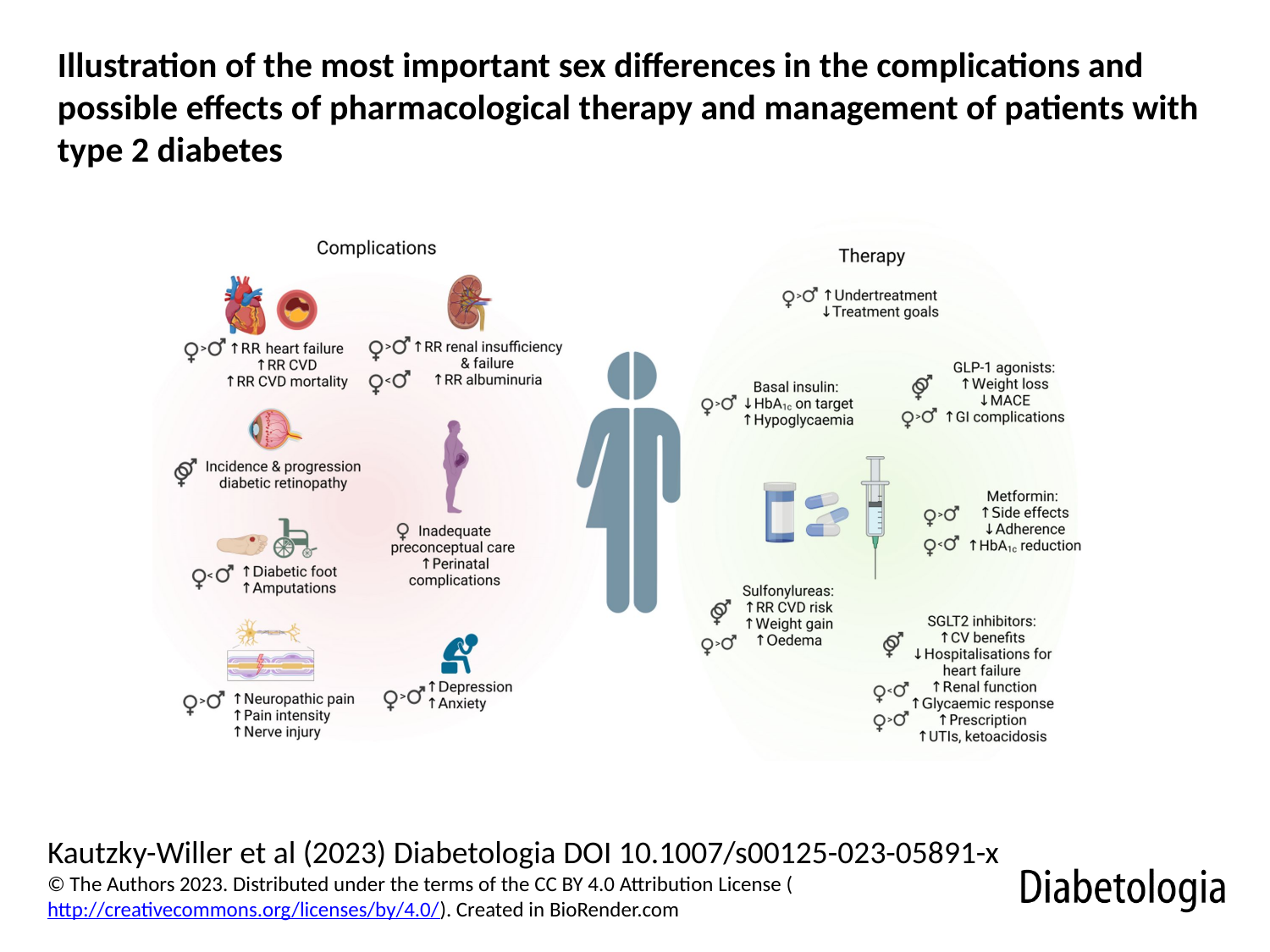

Illustration of the most important sex differences in the complications and possible effects of pharmacological therapy and management of patients with type 2 diabetes
Kautzky-Willer et al (2023) Diabetologia DOI 10.1007/s00125-023-05891-x
© The Authors 2023. Distributed under the terms of the CC BY 4.0 Attribution License (http://creativecommons.org/licenses/by/4.0/). Created in BioRender.com
